# Association of Myopia with cognitive function among one million adolescents

**DOI:** 10.1186/s12889-020-08765-8

**Published:** 2020-05-08

**Authors:** Jacob Megreli, Adiel Barak, Maxim Bez, Dana Bez, Hagai Levine

**Affiliations:** 1 Medical Corps, Israel Defense Forces, Ramat-Gan, Israel; 2 Hebrew University-Hadassah Faculty of Medicine, Braun School of Public Health and Community Medicine, P.O Box 12272, 9112002 Jerusalem, Israel; 3 Department of Ophthalmology, Tel Aviv Sourasky Medical Center, Tel Aviv, Israel; 4 Sackler Faculty of Medicine, Tel Aviv University, Tel Aviv, Israel

**Keywords:** Myopia, Cognition, Cross-sectional study

## Abstract

**Background:**

Myopia is a leading cause of visual impairment worldwide, and its increasing incidence is of public health concern. Cognitive function was associated with myopia among children, but evidence for adolescents is scarce. The purpose of this study was to determine whether myopia is associated with cognitive function, and which cognitive ability, verbal or non-verbal, is involved.

**Methods:**

We conducted a population-based cross-sectional study of 1,022,425 Israeli candidates for military service aged 16.5–18 years. Participants underwent a comprehensive battery of tests assessing verbal and non-verbal intelligence, which yields a summarized cognitive function score (CFS). In addition, subjective visual acuity examination followed by objective non-cycloplegic refraction was carried out for each participant. Association between myopia and cognitive function was evaluated by multivariable logistic regression models adjusted for gender, age, country of origin, socioeconomic status, years of education, body mass index, height and year of examination.

**Results:**

Compared to the intermediate CFS of the entire cohort, participants who had the highest CFS had 1.85-fold (95% CI, 1.81 to 1.89; *P* < .001) higher odds of having myopia and 2.73-fold (95% CI, 2.58 to 2.88; *P* < .001) higher odds of high myopia, while participants with the lowest CFS had 0.59-fold (95% CI, 0.57 to 0.61, *P* < .001) lower odds of having myopia. The verbal components of the cognitive function assessment had stronger associations with myopia than the non-verbal components (*P* < .001, for all).

**Conclusions:**

Cognitive function, especially verbal intelligence, is strongly and consistently associated with myopia among adolescents.

## Background

Myopia is a functional and medical problem, and has become an emerging public health problem during the last several decades [1–4]. Myopia generally occurs as a result of axial elongation of the eyeball during childhood. It is currently estimated that nearly 23% of the world’s population has myopia, a figure which is expected to double by 2050 [5]. Myopia, and especially high myopia, often results in serious problems that can lead to detrimental ramifications, including glaucoma, macular degeneration, detachment of retina, and cataract [6].

Myopia is a multifactorial disorder that is currently considered to be affected by both environmental and hereditary factors [7–9]. Cognitive function is one of the most arguable and investigated associations with myopia [10, 11]. Some early works studying this association suggested that a pleiotropic relationship between high cognitive function and myopia may exist, whereby a single or a group of genes might be responsible for both traits [12]. While the mechanism of this association remains controversial, the majority of the studies exploring this association focused only on children, leading to a scarcity of literature among adolescents and young adults [10]. Only one study reported a significant association between myopia and cognitive performance in adults 40–79 years of age [13], while some studies found no statistically significant relationship between myopia and cognitive function [14–16].

The aim of this study was to examine the association between myopia and cognitive function, and the specific cognitive abilities involved – verbal and non-verbal, in order to direct further research on the development of myopia, its etiology, epidemiology and pathophysiology.

## Methods

### Study design and population

**Fig. 1:**
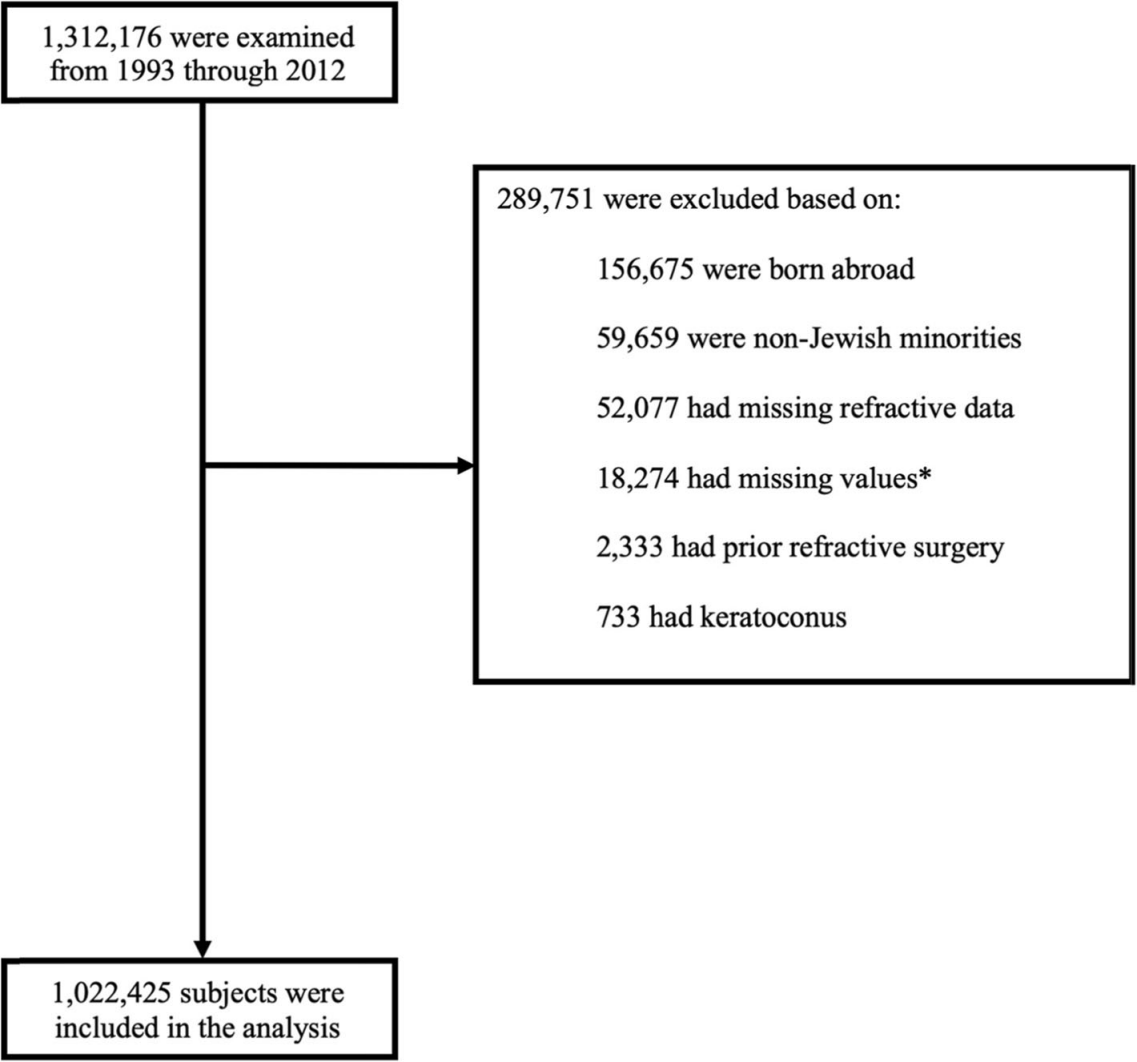
Flow diagram of selected study population. *Missing values: cognitive function score: 4553 (0.4%); country of origin: 7558 (0.6%); socioeconomic status: 4927 (0.4%); height: 384 (0.03%); body mass index: 642 (0.1%); years of education: 210 (0.02%)

Israeli adolescents recruited for mandatory military service undergo a medical and cognitive assessment at mean age of 17 years. We performed a population-based cross-sectional study, based on the nationwide Israel Defense Forces (IDF) conscription registry from 1993 to 2012. We limited the study till 2012, as since 2013, the eligible population for assessment changed to include the ultra-Orthodox Jewish population, who have extremely high proportions of myopia (82%) [17]. Total of 1,312,176 adolescents between 16.5 to 18 years of age were examined by the draft board (Fig. 1). We excluded 52,077 recruits with missing refractive measurements; 2333 who had prior refractive surgery; 733 with keratoconus; 18,274 with missing cognitive assessment, socio-demographic or anthropometric information; 59,659 from a non-Jewish population, since they are largely exempt from military service with only a small fraction being called up for a medical evaluation, thereby these adolescents were not representative of the overall minority population [18]; 156,675 who were born abroad, since the tests assessing cognitive function are administrated only in Hebrew therefore it might create a language barrier, and because the prevalence of myopia varies across populations of different geographic regions [3]. These exclusions resulted in a study sample of 1,022,425 participants. This study was approved by the Institutional Ethics Committee of the Israel Defense Forces Medical Corps (Approval No. 1669–2016) and conformed to the tenets of the Declaration of Helsinki. Participants anonymity was preserved. Patient consent was waived as the raw data was deidentified. Authors had full access to the database of the IDF Medical Corps, located at the Surgeon General’s headquarters in Ramat-Gan, Israel.

### Visual acuity examination

Visual acuity examination by a standard Snellen chart was carried out for each participant by a qualified technician. The candidate’s unaided visual acuity was evaluated using a standard Snellen chart at 6 m. All candidates with unaided visual acuity lower than 6/6 m underwent an objective non-cycloplegic refraction with an Autorefractometer (Speedy K; Nikon Corp., Tokyo, Japan; KR-8000, KR7000S and earlier models, Topcon, Tokyo, Japan) [19], followed by a complementary subjective refraction, for validation, using a standard Snellen chart. For each candidate, spherical equivalent (SEQ) was calculated separately for each eye according to the following formula: (SEQ = sphere power + [cylinder power/2]) [20]. Myopia was defined as SEQ of − 0.50 diopter (D) or less. Low myopia was defined as an SEQ between − 0.50 and − 2.99 D, moderate myopia was defined as an SEQ between − 3.00 and − 5.99 D, and high myopia was defined as an SEQ of − 6.00 D or less. The classification for each individual was made based on the worse SEQ between both eyes. Worse SEQ was chosen to be used for myopia definitions after preliminary analysis that demonstrated a satisfactory correlation between both eyes (Pearson correlation coefficient, 0.92) [21, 22].

### Cognitive assessment

Participants underwent a comprehensive cognitive assessment by a battery of tests that yields a cognitive function score (CFS) [23]. The score is normally distributed in the population, ranging from one to nine, so an intermediate CFS of five was considered the reference group. CFS is considered a valid measure of general intelligence, and is highly correlated with the *Wechsler* Adult Intelligence Scale [23–25]. The battery of cognitive assessment tests was composed of four subtests: two subtests assessed verbal intelligence (similarities and verbal instructions tests), and two subtests assessed non-verbal intelligence (arithmetic and visual-spatial tests), as previously described by Rabinowitz et al. [25]. These four subtests were classified separately into eight percentile groups (< 5th: reference group; 5-10th; 10-25th; 25-50th; 50-75th; 75-90th; 90-95th; > 95th). In order to enable comparisons between the four subtests, they were also classified by median, with the lower half being the reference group.

### Covariates and study variables

Socio-demographic and anthropometric data were recorded as part of the draft board intake process. Education was grouped according to number of years of formal schooling: < 9, 10 to 11, and > 12 (which includes higher and academic studies). Socioeconomic status (SES) was determined according to the Israeli Ministry of Interior classification, based on city of residence [26], as low, medium or high. Place of origin was determined by the birthplace of the father or grandfather (if the father was Israeli-born), and categorized according to country of origin. Body mass index (BMI) was calculated as weight in kilograms divided by height in meters squared. BMI and height values were coded according to the age and sex adjusted growth charts of the United States Centers for Disease Control and Prevention (CDC) [27].

### Statistical analysis

Univariable logistic regression models were used to evaluate the associations between myopia, as an outcome variable, to each of the dependent variables and covariates. The χ^2^ test was used for categorical variables, and 1-way analysis of variance was used for continuous variables. The association between CFS and myopia was assessed using multivariable logistic regression models, adjusted for all predetermined covariates that were found to be statistically significant in the univariable regression models: age (continuous variable), gender, country of origin, SES, years of education, BMI, height, and year of examination (ordinal variable). Same multivariable regression models were used separately to estimate the association of the four cognitive assessment subtests to myopia. Two-sided *P* < .001 was considered statistically significant. Results of the regression models are reported as odds ratio (OR) with 95% confidence interval (95% CI). Analyses were performed using SPSS statistics for Windows version 22 (IBM, Armonk, NY, USA).

## Results

Characteristics of the study participants are described in Table 1. The mean age of the 1,022,425 participants at the time of evaluation was 17.2 ± 0.3 years, 569,551 (55.7%) of them were males, the majority had completed high school, and more than 50% were classified as medium socioeconomic status. The overall prevalence of myopia among the study participants was 32.2%: 19.9% had mild myopia, 9.4% had moderate myopia, and 2.9% had high myopia. Myopia was more prevalent among females than males: 34.2% vs 30.6%, respectively (*P* < .001). Prevalence of myopia was lower among immigrants from Ethiopia (19.0%). Both immigrants from Ethiopia and former USSR countries had large-scale immigration waves at more recent times compared to the rest of the population. There was a significant increase in the prevalence of myopia during the 20-year period of examination: from 26.9% in 1993 to 33% in 2012 (*P* < .001; eTable 1 in the Supplement). Univariable analysis showed that age, gender, country of origin, SES, years of education, BMI, height and year of examination were all associated with myopia (*P* < .001).

**Table 1:** Myopia Prevalence by Sociodemographic Variables, Univariable Logistic Regression Analysis

Variable		Adolescents with Myopia, No./Total No. (%)	***P*** Value
Gender	Female	154857/452874 (34.2)	<.001
	Male	174016/569551 (30.6)	
Country of origin	Western	98543/296049 (33.3)	<.001
	North-African	81563/278154 (29.3)	
	Asia	92034/268265 (34.3)	
	Former USSR	26481/82488 (32.1)	
	Ethiopia	1697/8927 (19.0)	
	Israel	28555/88542 (32.3)	
Socioeconomic status^a^	Low	81185/235072 (34.5)	<.001
	Medium	169265/546684 (31.0)	
	High	78423/240669 (32.6)	
Years of education	< 9	2478/12793 (19.4)	<.001
	10–11	13632/53825 (25.3)	
	> 12	312763/955807 (32.7)	
BMI^b^	Underweight	33874/100070 (33.9)	<.001
	Normal weight	250232/787254 (31.8)	
	Overweight	28127/86113 (32.7)	
	Obese	16640/48988 (34.0)	
Height^c^	Short	19128/54717 (35.0)	<.001
	Normal	298836/934926 (32.0)	
	Tall	10909/32782 (33.3)	
Total		328873/1022425 (32.2)	

*Abbreviations*: *USSR* Union of Soviet Socialist Republics, *BMI* Body mass index

^a^According to the Israeli Ministry of Interior classification: low (1st-4th deciles), medium (5th–7th deciles) and high (8th–10th deciles)

^b^Body mass index. Sex- and Age- (by months) adjusted percentiles of BMI and height according to the United States Centers for Disease Control and Prevention (CDC) 2000 growth charts. BMI classification: underweight (BMI <5th percentile), normal weight (5th percentile < BMI < 85th), overweight (85th percentile < BMI < 95th), and obese (BMI > 95th percentile)

^c^Height classification: short (Height < 5th percentile), normal (5th percentile < Height < 95th), and tall (Height > 95th percentile)

**Fig. 2:**
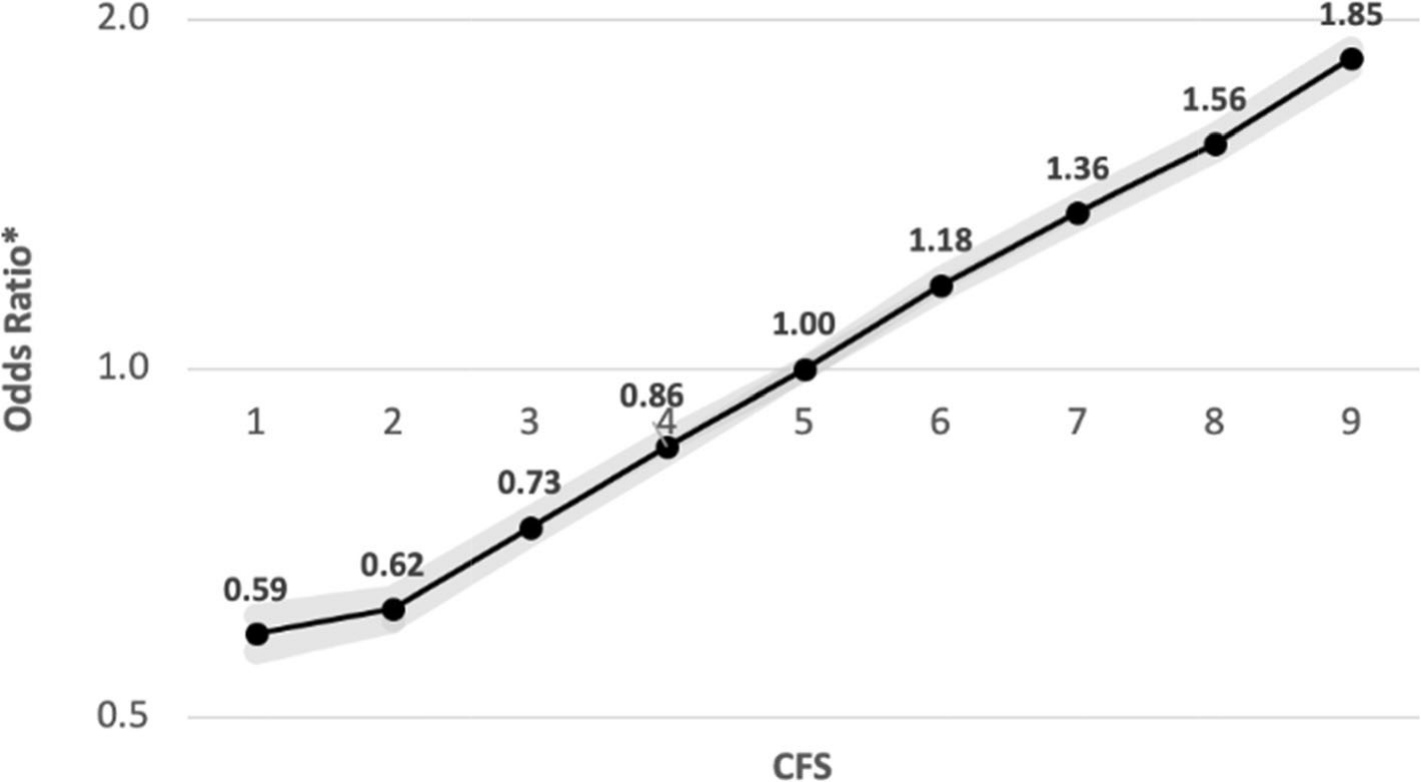
Association of cognitive function score with myopia. Values represent the odds ratio for each CFS group in comparison with the intermediate CFS. Gray area represents the 95% confidence interval. Adjusted odds ratio for gender, age, country of origin, socioeconomic status, years of education, BMI, height and year of examination by multivariable logistic regression model. *P < .001 for all comparisons (*N* = 1,022,425)

**Fig. 3:**
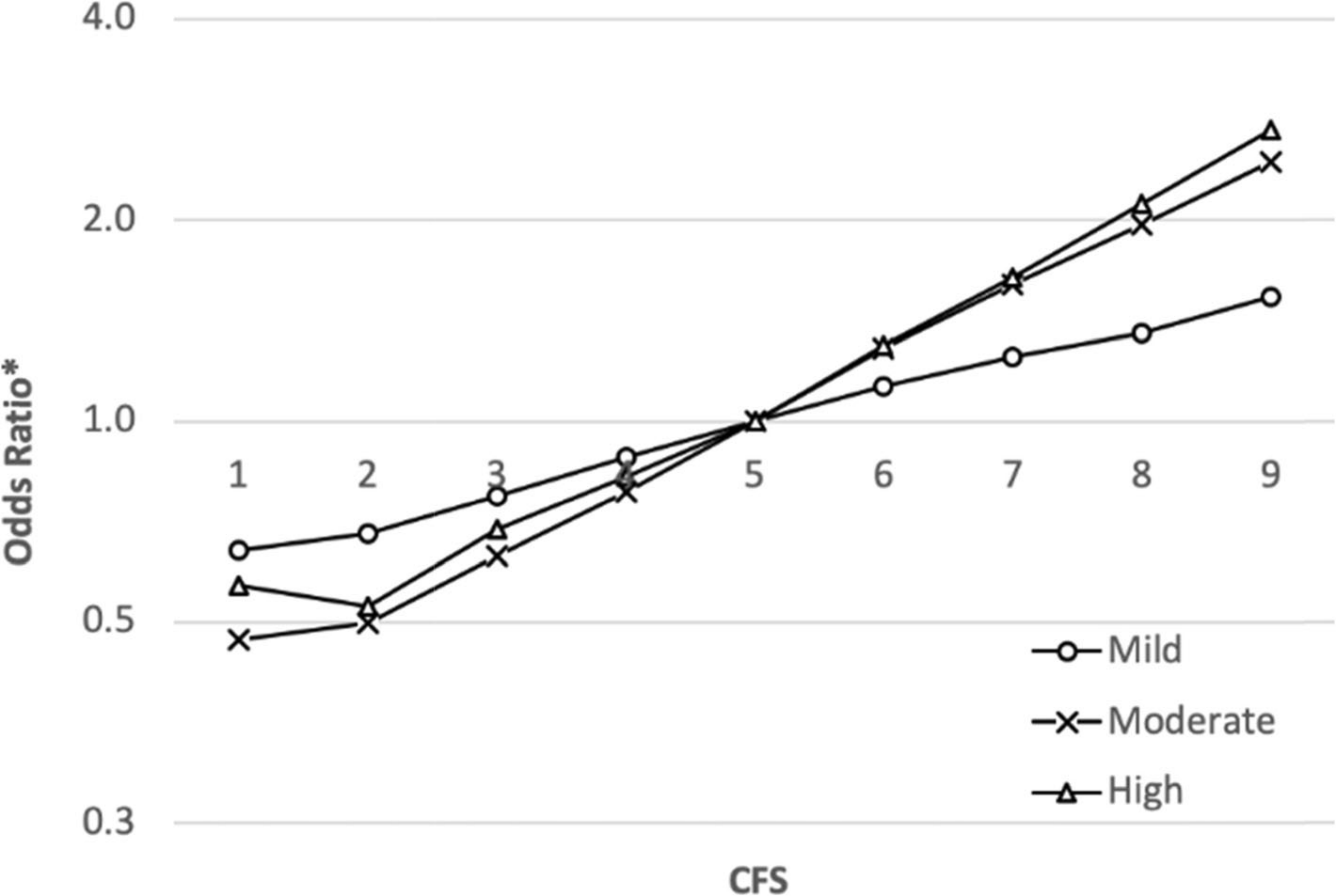
Association of cognitive function score with mild, moderate and high myopia. Adjusted odds ratio for gender, age, country of origin, socioeconomic status, years of education, BMI, height and year of examination by multivariable logistic regression model for each severity of myopia independently. *P < .001 for all comparisons (*N* = 1,022,425)

The prevalence of myopia increased gradually in accordance with CFS: from 21.7% among those with the lowest score to 43.3% among those with the highest score (*P* < .001; Table 2). CFS was found to have a consistent positive association with myopia in univariable regression models. Following multivariable adjustment, the association between CFS and myopia remained consistent and became accentuated (Fig. 2). Compared to the intermediate CFS, participants with the highest CFS had 1.85-fold (95% CI, 1.81 to 1.89; *P* < .001) higher odds of having myopia, while participants with the lowest CFS had 0.59-fold (95% CI, 0.57 to 0.61, *P* < .001) lower odds of having myopia. Association between CFS and myopia remained consistent regardless of the severity of the myopia, and was found to be the strongest for moderate and high myopia (Fig. 3). In comparison with the intermediate CFS, participants who had the highest CFS had 1.54-fold (95% CI, 1.50 to 1.58; *P* < .001) higher odds of having mild myopia, 2.45-fold (95% CI, 2.37 to 2.53; *P* < .001) higher odds of having moderate myopia, and 2.73-fold (95% CI, 2.58 to 2.88; *P* < .001) higher odds of having high myopia.

**Table 2:** Cognitive characteristics and association with myopia in univariable and multivariable models

CFS	Adolescents with Myopia, No./Total No. (%)	Unadjusted OR (95% CI)	Adjusted OR^**a**^ (95% CI)
**1**	4619/21319 (21.7)	0.61 (0.59–0.63)	0.59 (0.57–0.61)
**2**	10845/47825 (22.7)	0.65 (0.64–0.67)	0.61 (0.61–0.64)
**3**	25988/101698 (25.6)	0.76 (0.75–0.77)	0.72 (0.72–0.74)
**4**	47062/166015 (28.3)	0.87 (0.86–0.89)	0.85 (0.84–0.87)
**5**	65860/211885 (31.1)	1.00 [Reference]	
**6**	69023/202101 (34.2)	1.14 (1.14–1.17)	1.18 (1.17–1.20)
**7**	53842/145595 (37.0)	1.30 (1.28–1.32)	1.36 (1.34–1.38)
**8**	32503/81779 (39.7)	1.46 (1.44–1.49)	1.56 (1.54–1.59)
**9**	19131/44208 (43.3)	1.69 (1.66–1.73)	1.85 (1.81–1.89)

*OR* Odds ratio, *CI* Confidence interval, *CFS* Cognitive function score

^a^Adjusted odds ratio for age, gender, country of origin, socioeconomic status, years of education, BMI, height and year of examination by multivariable logistic regression model

*P* value was <.001 for all comparisons, both unadjusted and adjusted

**Fig. 4:**
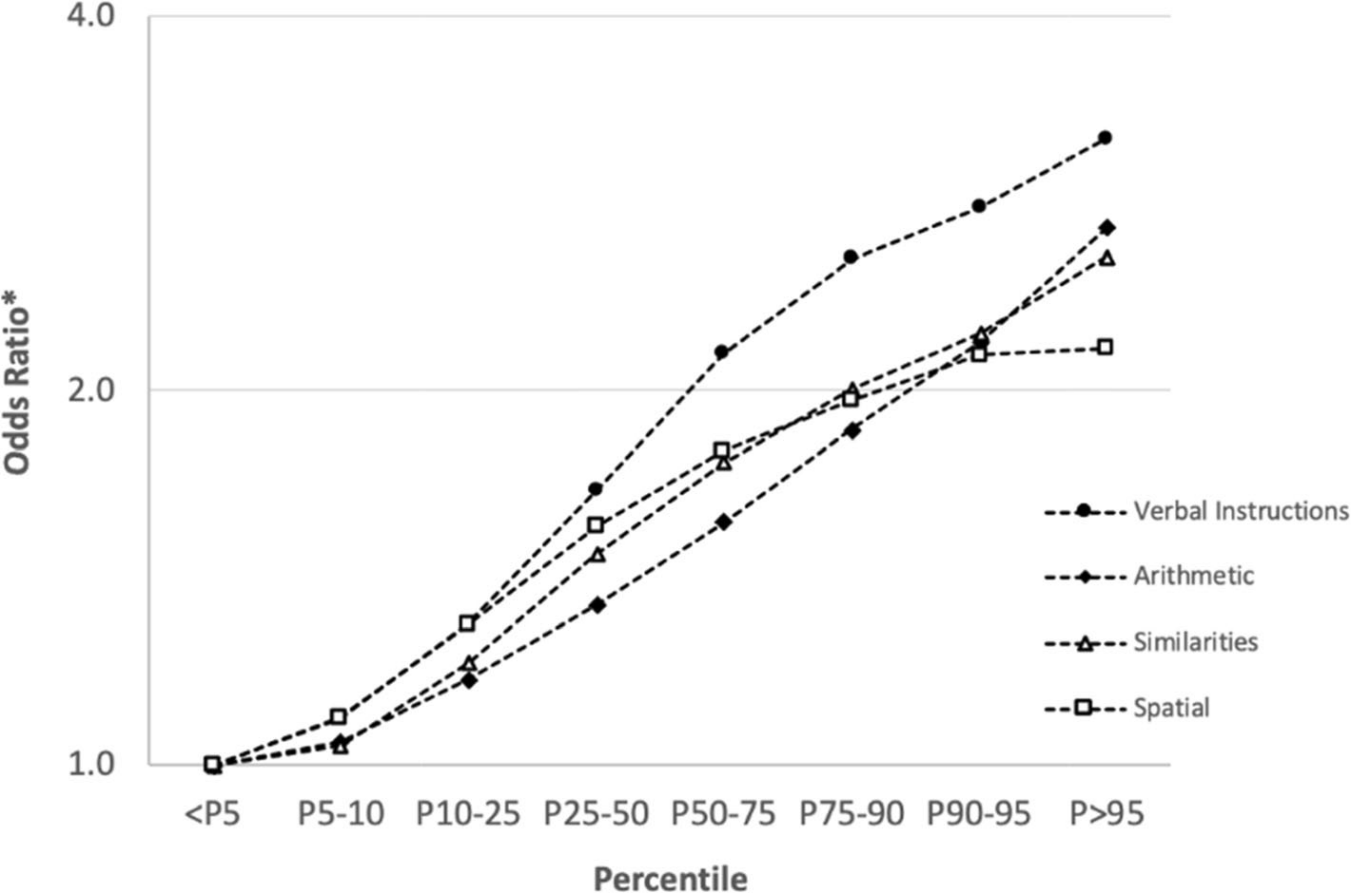
Association of cognitive function subtests with myopia in multivariable models. Values represent the odds ratio for myopia in the 95th percentile group compared to the <5th percentile group in each subtest. Adjusted odds ratio for gender, age, country of origin, socioeconomic status, years of education, BMI, height and year of examination by multivariable logistic regression model for each subtest independently. *P < .001 for all comparisons (*N* = 1,022,425)

Both the verbal and non-verbal components of the CFS had a consistent positive association with myopia in univariable regression models (eTable 2 in the Supplement). Following multivariable adjustment, the association with myopia in each of the subtests was further accentuated and remained consistent (Fig. 4). Markedly, the verbal instructions subtest had the strongest association with myopia (OR, 3.19; 95% CI, 3.10 to 3.28; *P* < .001).

The consistent positive association found between CFS and myopia persisted in a series of sensitivity analyses, including a sex-specific analysis (eTable 3 in the Supplement), and an analysis modeling the association with stricter definition of myopia (SEQ < − 1.00 diopter) (eFigure 1 in the Supplement). In our population, only 6.5% had less than 12 years of education. In a stratified analysis by years of education, the association between CFS and myopia persisted across strata. The confidence interval was wide for those with lower years of education and high CFS, due to small number of participants (eTable 4**)**.

## Discussion

In this comprehensive study among 1,022,425 Israeli adolescents, we found cogent evidence that cognitive function is strongly and consistently associated with myopia, independent of age, gender, country of origin, socioeconomic status, years of education, body mass index, height and year of examination. Both the verbal and non-verbal components of the cognitive evaluation were associated with myopia.

Myopia is considered a multifactorial disorder that is affected by both hereditary and environmental factors [7–9]. Cognitive function was assumed to be one of the most arguable and investigated associations with myopia [10]. Several studies found a significantly positive association between cognitive function and myopia among children [28], Jewish-adolescent males [29], and among adults and the elderly [13, 30], while others found lack of statistically significant relationship [14–16]. In this study we found a positive, strong and independent association between cognitive function and myopia, which was consistent regardless of the severity of the myopia or the cognitive subtest.

The mechanism of the association between intelligence and myopia remains to be clarified [31]. One of the plausible explanations is a behavioral relationship between the two traits [32]. It was previously reported that subjects who read more or engage in educational activities have superior performance on intelligence tests, particularly those assessing verbal intelligence [33]. Additional works have shown that greater amount of near-work activity such as reading increases the odds of having myopia [20, 34]. Bez et al. [17] has recently reported a 9.3-fold increased odds of having myopia among ultra-Orthodox Jewish students exposed to near-work activities from a very young age compared with age-matched secular students. Similarly, another study found significantly higher rates of myopia among Orthodox Jewish male students, emphasizing the effect that near-work activities have on the development of myopia [35]. This argument gains support from our finding that verbal intelligence tests, which require acquisition of linguistic skills mainly through reading, had stronger association with myopia than non-verbal intelligence tests. On the other hand, one can argue that the amount of reading is determined by the level of a person’s intelligence, and therefore there is a predisposition among more intelligent children to develop myopia. This notion is countered by our finding of a statistically significant association between myopia and spatial intelligence, which was assessed by a modified version of Raven’s Progressive Matrices [25]. This test, which examines visual-spatial problem solving abilities, does not rely on linguistic abilities or previously acquired information [36]. This finding also counters the argument by Young et al. that myopes have higher scores on intelligence tests because they are more capable of quick and efficient reading than emmetropes or hypermetropes [14, 37].

Another possible, yet unlikely explanation for the association between intelligence and myopia rests on a biological relationship, according to which myopia is an overdevelopment of the eye, and as ocular and cerebral development are related [31], this in turn leads to superior intelligence among myopes [38]. Some researchers suggested that there is a pleiotropic relationship between myopia and cognitive function, i.e. both traits are affected by the same gene or set of genes [12, 39, 40]. Several reviews covered the genes associated with myopia [41–43], but none addressed the association of these genes with cognitive development. More recent genome-wide association studies found a modest but significant contribution of pleiotropic genetic factors contributing to the development of myopia and higher intelligence [44]. However, based on the current evidence, there are no genes that were proven to play a major role in the development of these traits.

This study has several limitations. First, a causal inference between cognitive function and myopia cannot be established from a cross-sectional design. Second, the database had no data on the refractive error of the subjects’ parents, which might be relevant for the genetic component of myopia [45]. The rapid increase in myopia prevalence during the past several decades that was also observed in our analysis does, however, give weight to the crucial influence of environmental factors in the development of myopia. Third, our sample is not necessarily representing the Israeli female adolescent population, as approximately 30% of them are not recruited to the IDF mainly because of religious beliefs [46], therefore the association between cognitive function and myopia among the female population should be interpreted with more caution. Fourth, the non-cycloplegic refraction method used in this study, which results in a slight overestimation of myopia, especially among children and young adults [47], is less accurate than cycloplegic refraction. It was previously shown that non-cycloplegic refraction can overestimate the prevalence of myopia in populations up to the age of 50 and that cycloplegic refraction should be used in these age groups [48]. Nevertheless, using a stricter definition for myopia (SEQ < -1.00 diopter) reaffirms the main association found in this study (eFigure 1 in the Supplement). Our SES variable was crude as the classification was made according to the participants’ settlements. This may explain the weak association found between SES and prevalence of myopia. Lastly, we included conscripts examined up to the year 2012 due to change in draft policy that included the enlistment of the ultra-Orthodox Jewish population, who have extremely high proportions of myopia (82%) [17]. Future studies will analyze the association in newer cohorts.

## Conclusions

In conclusion, we found cogent evidence of the association of cognitive function with myopia in more than one million Israeli adolescents. This finding was most pronounced for verbal intelligence subtests, which require acquisition of linguistic skills. Findings of our study point to the role of educational activity and intensive reading in the development of myopia. Further research is warranted to replicate these findings in other populations and study its mechanism.

## Supplementary information

**Additional file 1: eTable 1.** Myopia prevalence by year of examination, univariable logistic regression analysis. **eTable 2.** Cognitive function assessment subtests and association with myopia in univariable and multivariable models. **eTable 3.** Association of cognitive function score (CFS) with myopia among males and females in multivariable models. **eTable 4.** Association of cognitive function score (CFS) with myopia with stratification for years of education in multivariable models. **eFigure 1.** Sensitivity analysis of the association of cognitive function score (CFS) with stricter definition of myopia (<− 1.00 Diopter). Values represent the odds ratio for each CFS group in comparison with the intermediate CFS. Gray area represents the 95% confidence interval. *P* < .001 for all comparisons. Adjusted odds ratio for age, gender, country of origin, socioeconomic status, years of education, BMI, height and year of examination by multivariable logistic regression model. (CFS, cognitive function score).

## Data Availability

The datasets used and/or analysed during the current study are available from the corresponding author on reasonable request.

## Abbreviations

IDF: Israel Defense Forces
BCVA: Best-corrected visual acuity
SEQ: Spherical equivalent
CFS: Cognitive function score
BMI: Body mass index
SES: Socioeconomic status
CDC: Center for Disease Control
OR: Odds ratio
CI: Confidence interval
