# Supplementary material for: Association of Myopia with cognitive function among one million adolescents
Source: BMC Public Health. 2020 May 8;20:647. doi: 10.1186/s12889-020-08765-8 (PMC7206693; doi:10.1186/s12889-020-08765-8)
Supplement: Supplementary file 1 — Additional file 1: eTable 1. Myopia prevalence by year of examination, univariable logistic regression analysis. eTable 2. Cognitive function assessment subtests and association with myopia in univariable and multivariable models. eTable 3. Association of cognitive function score (CFS) with myopia among males and females in multivariable models. eTable 4. Association of cognitive function score (CFS) with myopia with stratification for years of education in multivariable models. eFigure 1. Sensitivity analysis of the association of cognitive function score (CFS) with stricter definition of myopia (<− 1.00 Diopter). Values represent the odds ratio for each CFS group in comparison with the intermediate CFS. Gray area represents the 95% confidence interval. P < .001 for all comparisons. Adjusted odds ratio for age, gender, country of origin, socioeconomic status, years of education, BMI, height and year of examination by multivariable logistic regression model. (CFS, cognitive function score). [file 12889_2020_8765_MOESM1_ESM.pdf]

eTable 1. Myopia prevalence by year of examination, univariable logistic regression analysis

Year of Examination	Adolescents with Myopia, No./Total No. (%)	OR (95% CI)	P Value
1993	16267/60536 (26.9)	1 [Reference]	
1994	15173/55339 (27.4)	1.028 (1.002-1.05)	.037
1995	15486/53872 (28.7)	1.098 (1.07-1.13)	<.001
1996	16919/59286 (28.5)	1.087 (1.06-1.11)	
1997	13774/46921 (29.4)	1.131 (1.10-1.16)	
1998	18028/55600 (32.4)	1.306 (1.27-1.34)	
1999	20040/58653 (34.2)	1.412 (1.38-1.45)	
2000	17501/51529 (34.0)	1.400 (1.36-1.44)	
2001	19575/57140 (34.3)	1.418 (1.38-1.45)	
2002	18343/56172 (32.7)	1.320 (1.29-1.35)	
2003	18088/52457 (34.5)	1.432 (1.39-1.47)	
2004	17478/50328 (34.7)	1.448 (1.41-1.49)	
2005	17425/51906 (33.6)	1.375 (1.34-1.41)	
2006	18624/54654 (34.1)	1.407 (1.37-1.44)	
2007	17895/51968 (34.4)	1.429 (1.39-1.47)	
2008	16174/46638 (34.7)	1.445 (1.41-1.48)	
2009	19524/57411 (34.0)	1.402 (1.37-1.44)	
2010	18643/54711 (34.1)	1.407 (1.37-1.44)	
2011	15957/49659 (32.1)	1.289 (1.25-1.32)	
2012	19742/59793 (33.0)	1.341 (1.31-1.37)	

eTable 2. Cognitive function assessment subtests and association with myopia in univariable and multivariable models

Cognitive Assessment Subtest percentile	Unadjusted OR (95% CI)	Adjusted OR[a] (95% CI)	P value for unadjusted	P value for adjusted
**Verbal Instructions**				
<P5	1.00 [Reference]			
P5-10	1.09 (1.05-1.13)	1.09 (1.05-1.13)	<.001	<.001
P10-25	1.24 (1.20-1.27)	1.30 (1.26-1.34)		
P25-50	1.54 (1.50-1.58)	1.66 (1.62-1.71)		
P50-75	1.91 (1.87-1.96)	2.14 (2.09-2.20)		
P75-90	2.26 (2.20-2.32)	2.55 (2.48-2.62)		
P90-95	2.50 (2.43-2.57)	2.81 (2.73-2.89)		
P>95	2.87 (2.79-2.95)	3.19 (3.10-3.28)		
**Similarities**				
<P5	1.00 [Reference]			
P5-10	1.03 (1.00-1.06)	1.04 (1.01-1.07)	.043	.019
P10-25	1.19 (1.16-1.22)	1.21 (1.18-1.24)	<.001	<.001
P25-50	1.44 (1.40-1.47)	1.48 (1.44-1.51)		
P50-75	1.68 (1.64-1.72)	1.75 (1.71-1.79)		
P75-90	1.90 (1.85-1.94)	2.00 (1.95-2.06)		
P90-95	2.08 (2.03-2.14)	2.22 (2.16-2.29)		
P>95	2.36 (2.30-2.42)	2.57 (2.50-2.64)		
**Arithmetic**				
<P5	1.00 [Reference]			
P5-10	1.04 (1.00-1.08)	1.04 (1.01-1.08)	.029	.021
P10-25	1.15 (1.12-1.18)	1.17 (1.14-1.21)	<.001	<.001
P25-50	1.28 (1.24-1.31)	1.35 (1.31-1.38)		
P50-75	1.42 (1.38-1.46)	1.57 (1.53-1.61)		
P75-90	1.62 (1.58-1.67)	1.86 (1.81-1.91)		
P90-95	1.85 (1.80-1.91)	2.19 (2.12-2.26)		
P>95	2.21 (2.14-2.28)	2.70 (2.62-2.79)		
**Spatial**				
<P5	1.00 [Reference]			
P5-10	1.09 (1.06-1.12)	1.09 (1.06-1.13)	<.001	<.001
P10-25	1.28 (1.25-1.31)	1.30 (1.27-1.34)		
P25-50	1.51 (1.48-1.55)	1.56 (1.52-1.60)		
P50-75	1.72 (1.68-1.77)	1.79 (1.75-1.84)		
P75-90	1.88 (1.83-1.92)	1.96 (1.91-2.02)		
P90-95	2.03 (1.98-2.09)	2.14 (2.08-2.20)		
P>95	2.05 (1.99-2.11)	2.16 (2.10-2.23)		

[a] Adjusted odds ratio for gender, age, country of origin, socioeconomic status, years of education, BMI, height and year of examination by multivariable logistic regression model.

CI: confidence interval;

eTable 3. Association of cognitive function score (CFS) with myopia among males and females in multivariable models

CFS	Males (N=569,551)		Females (N=452,874)	
	**Adjusted OR[a] (95% CI)**	**P value**	**Adjusted OR[a] (95% CI)**	**P value**
**1**	0.61 (0.59-0.64)	<.001	0.51 (0.48-0.55)	<.001
**2**	0.63 (0.61-0.65)		0.60 (0.57-0.62)	
**3**	0.73 (0.71-0.74)		0.73 (0.71-0.75)	
**4**	0.85 (0.83-0.87)		0.86 (0.84-0.88)	
**5**	1.00 [Reference]		1.00 [Reference]	
**6**	1.20 (1.17-1.22)	<.001	1.17 (1.15-1.19)	<.001
**7**	1.40 (1.37-1.43)		1.33 (1.30-1.36)	
**8**	1.63 (1.59-1.66)		1.49 (1.46-1.54)	
**9**	1.95 (1.90-2.00)		1.72 (1.66-1.79)	

[a] Adjusted odds ratio for age, country of origin, socioeconomic status, years of education, BMI, height and year of examination by multivariable logistic regression model.

CI: confidence interval; CFS: cognitive function score;

eTable 4. Association of cognitive function score (CFS) with myopia with stratification for years of education in multivariable models

CFS	≤9 years (N=12,793)		10-11 years (N=53,825)		≥12 years (N=955,807)	
	**Adjusted OR[a] (95% CI)**	**P-value**	**Adjusted OR[a] (95% CI)**	**P-value**	**Adjusted OR[a] (95% CI)**	**P-value**
**1**	0.59 (0.49-0.71)	<.001	0.66 (0.61-0.72)	<.001	0.57 (0.54-0.59)	<.001
**2**	0.67 (0.56-0.80)	<.001	0.70 (0.65-0.75)	<.001	0.61 (0.59-0.62)	<.001
**3**	0.73 (0.61-0.87)	<.001	0.76 (0.71-0.81)	<.001	0.73 (0.72-0.75)	<.001
**4**	0.90 (0.75-1.08)	.27	0.86 (0.80-0.92)	<.001	0.86 (0.85-0.87)	<.001
**5**	1.00 [Reference]		1.00 [Reference]		1.00 [Reference]	
**6**	1.44 (1.11-1.88)	.007	1.17 (1.07-1.27)	<.001	1.18 (1.17-1.30)	<.001
**7**	1.41 (0.97-2.05)	.075	1.23 (1.10-1.38)	<.001	1.37 (1.35-1.39)	<.001
**8**	2.20 (1.16-4.15)	.015	1.41 (1.20-1.67)	<.001	1.57 (1.54-1.60)	<.001
**9**	2.46 (0.92-6.59)	.072	1.29 (1.00-1.67)	.046	1.87 (1.83-1.91)	<.001

[a] Adjusted odds ratio for age, gender, country of origin, socioeconomic status, BMI, height and year of examination by multivariable logistic regression model.

CI: confidence interval; CFS: cognitive function score;

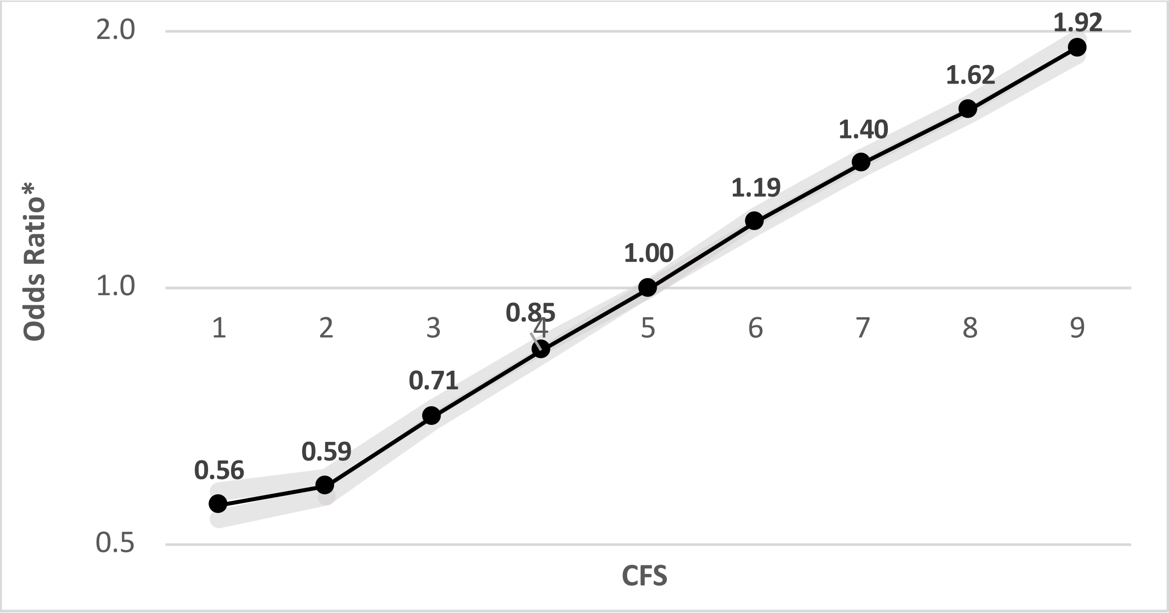

eFigure 1. Sensitivity analysis of the association of cognitive function score (CFS) with stricter definition of myopia (≤-1.00 Diopter). Values represent the odds ratio for each CFS group in comparison with the intermediate CFS. Gray area represents the 95% confidence interval. *P*<.001 for all comparisons. Adjusted odds ratio for age, gender, country of origin, socioeconomic status, years of education, BMI, height and year of examination by multivariable logistic regression model. (CFS, cognitive function score).
